# Semaphorin 4D correlates with increased bone resorption, hypercalcemia, and disease stage in newly diagnosed patients with multiple myeloma

**DOI:** 10.1038/s41408-018-0075-6

**Published:** 2018-05-11

**Authors:** Evangelos Terpos, Ioannis Ntanasis-Stathopoulos, Dimitrios Christoulas, Tina Bagratuni, Marios Bakogeorgos, Maria Gavriatopoulou, Evangelos Eleutherakis-Papaiakovou, Nikolaos Kanellias, Efstathios Kastritis, Meletios A. Dimopoulos

**Affiliations:** Department of Clinical Therapeutics, Alexandra General Hospital, Medical School, National and Kapodistrian University of Athens, Athens, Greece

## Abstract

Multiple myeloma (MM) is characterized by bone destruction due to increased bone resorption and decreased bone formation. Semaphorin 4D (CD100, Sema4D) is expressed by osteoclasts, binds to its receptor Plexin-B1, and acts as a mediator of osteoclast–osteoblast interaction that ultimately inhibits osteoblastic bone formation. Preclinical data suggest that Sema4D/Plexin-B1 pathway is implicated in MM-induced bone disease. However, there is no information on the role of Sema4D in MM patients. Thus, we evaluated Sema4D and Plexin-B1 in six myeloma cells lines in vitro; in the bone marrow plasma (BMP) and serum of 72 newly diagnosed symptomatic MM (NDMM) patients and in 25 healthy controls. Only one myeloma cell line produced high Sema4D. BMP and circulating Sema4D and Plexin-B1 levels were significantly higher in MM patients compared to controls (*p* < 0.01). Sema4D correlated with serum calcium levels (*p* < 0.001), increased bone resorption (as assessed by CTX; *p* < 0.01), and ISS (*p* < 0.001). There was a trend for higher Sema4D levels in patients with osteolysis (*p* = 0.07), while patients with diffuse MRI pattern had higher BMP Sema4D levels (*p* = 0.02). Our data suggest that Sema4D is elevated in MM patients and correlate with adverse myeloma features and increased bone resorption, providing a possible target for novel therapeutic approaches in MM.

## Introduction

Multiple myeloma (MM) is the second most common hematologic malignancy and is characterized by the development of osteolytic bone disease due to disrupted bone remodeling; there is an upregulation of bone resorption due to increased number and activity of osteoclasts, whereas bone formation is suppressed due to reduced osteoblast number and activity^1,2^. Semaphorins were originally described as axonal guidance molecules. However, they are also expressed outside the nervous system, in a wide variety of tissues, and are implicated in diverse biological procedures, including cell migration, immune response, tissue development, angiogenesis, and tumor progression^3,4^. Semaphorin 3A (Sema3A) is implicated as an antiangiogenic inducer in monoclonal gammopathies pathophysiology. More specifically, loss of Sema3A favors the angiogenic effect of Vascular Endothelial Growth Factor_165_ and may be involved in the angiogenic transition from monoclonal gammopathy of undetermined significance to MM^5^. Regarding bone metabolism, it has been suggested that semaphorins are involved in the cell–cell communication between osteoclasts and osteoblasts during bone remodeling^6,7^. Semaphorin 4D (CD100—Sema4D) is expressed by osteoclasts and acts as a mediator of osteoclast–osteoblast interaction that ultimately inhibits osteoblastic bone formation^8^. There are two known Sema4D receptors described: (a) CD72 in lymphoid and (b) Plexin-B1 in nonlymphoid cells^9–11^. Plexin-B1 expression is markedly induced during osteoblast differentiation^12^ and forms a receptor complex with either erythroblastic leukemia viral oncogene homolog 2 (ErbB2) or hepatocyte growth factor receptor (Met), depending on the cell type^13^. Binding of Semaphorin 4D to Plexin-B1 results in the phosphorylation of the kinase (ErbB2 or Met) and Plexin-B1^14^. In osteoblasts, expression of ErbB2 is higher than that of Met and Sema4D stimulation induces the phosphorylation of ErbB2, but not Met. Plexin-B1 functions as a receptor for Sema4D in osteoblasts and ErbB2 serves as an associating kinase. The semaphorin-plexin system regulates cell morphology and migration by modulation of actin cytoskeletal rearrangement, primarily via Rho family small GTPases^12^. Furthermore, activation of RhoA deregulates the IRS-1/IGF-1 signaling cascade that is essential for osteoblast differentiation^12^. Whereas the role of Sema4D and its receptor, Plexin-B1, complex in the bone remodeling process is established^8^, only limited preclinical evidence in myeloma-related osteolytic bone disease is available^15–17^. Therefore, the aim of our study was to investigate the potential role of this pathway in myeloma-related osteolytic bone disease.

## Materials and methods

First, we evaluated the Sema4D and Plexin-B1 production in the supernatant of myeloma cell lines and also by ovarian cancer cell lines. Then we scheduled a prospective study for the measurement of Sema4D and Plexin-B1 in myeloma patients.

### In vitro study design

#### Myeloma and ovarian cancer cell lines

We evaluated Sema4D and Plexin-B1 in the supernatants of six myeloma cell lines (LR5, MR20, L363, U261, H929, and JJN3) and four ovarian cancer cell lines (A2780, C30, OVCA3, and SKOV3) before and after incubation for 24 and 48 h with stromal cell line HS5 in order to establish osteogenic conditions. Ovarian cancer cell lines have shown increased Sema4D levels that may be associated with poor outcomes; thus, they have been chosen as comparators in the present study^18,19^.

All myeloma cell lines and ovarian cancer cell lines A2780 and C30 were maintained in RPMI-1640 (Biosera, UK) supplemented with 10% fetal bovine serum (FBS, Biosera). Bone stromal cell line (HS5) were maintained in Dulbecco’s Modified Eagle’s Medium (DMEM, Gibco) with 10% FBS. Ovarian cancer cells OVCA3 was maintained in RPMI-1640 with 20% FBS and SKOV3 was maintained in McCoy’s 5a Medium Modified (Gibco) supplemented with 10% FBS. All cells were propagated in standard cell culture conditions (5% CO_2_, 37 °C) in cell culture-treated T75 flasks. Media was replenished every 2–3 days. Once cells had reached 80–90% confluency, cells were split (1/3) in new T75 flasks.

#### Tumor-stromal co-cultures

Cultures of all cell lines (besides HS5) were plated at a density of ~60.000 cells per well in a 6-transwell plate. Plating density was chosen so that all cultures were confluent at the day of supernatant collection. Co-cultures containing HS5 cells were plated at a density of ~20.000 per well and maintained in 3:1 ratio (all cell lines:HS5 cells). The co-culture medium was also kept in 3:1 ratio (RPMI or McCoy’s:DMEM). To make sure that all cells had a normal growth curve at the above culture conditions, untreated cell lines (cells which were not co-cultured with HS5) were also grown at 3:1 RPMI or McCoy’s:DMEM solution. Media was carefully removed after 24 and 48 h, centrifuged for 5 min at 1000 rpm and the supernatant was collected for further analysis.

### Clinical study design

This was a prospective study for the evaluation of levels of Sema4D and Plexin-B1 in the serum and bone marrow plasma of myeloma patients and their correlation with features of the disease, including overall survival (OS).

#### Inclusion and exclusion criteria

The inclusion criteria of the study included: (i) adult patients with newly diagnosed symptomatic myeloma (NDMM) before the administration of any kind of therapy; (ii) patients who have given their written informed consent for blood sampling and for recording of their medical data which is pertinent to the purposes of this study.

The exclusion criteria included: (i) patients <18 years; (ii) presence of autoimmune disorders or other malignant diseases; (iii) use of medication that could alter the levels of the studied parameters (i.e., bisphosphonates) during the last 6 months before measurement.

#### Study endpoints

The primary endpoint of the study was the evaluation of serum levels of Sema4D and Plexin-B1 in NDMM at the time of diagnosis and their comparison with those of healthy individuals of similar age and gender.

Secondary endpoints included: (i) measurement of bone marrow plasma Sema4D and Plexin-B1 in NDMM; (ii) correlation of serum and bone marrow plasma Sema4D and Plexin-B1 with disease features (stage, osteolytic disease, bone markers, LDH, MRI pattern of marrow infiltration, etc.); (iii) correlation of serum and bone marrow plasma Sema4D and Plexin-B1 with survival; and (iv) correlation of serum and bone marrow plasma Sema4D and Plexin-B1with markers of bone metabolism, i.e., C-terminal cross-linking telopeptide of collagen type-I (CTX; a marker of bone resorption) and bone-specific alkaline phosphatase (bALP; a marker of bone formation).

#### Patients’ enrollment

The enrollment period was between January 2009 and January 2012. Patients were informed of the objectives and the details of the present study before they gave their approval and sign the informed consent form. The study was conducted according to the principles defined by the 18th World Medical Association Assembly (Declaration of Helsinki, 1964) and all its future amendments. The study protocol was designed and executed according to the guidelines and regulations pertaining to studies in Greece, as well as the Good Clinical Practice Guidelines as defined by the International Conference of Harmonization. The study was approved by the local ethics committee.

#### Control groups

In this study, circulating Sema4D and Plexin-B1 was also measured in the serum of 25, gender-matched and age-matched, healthy controls and in the bone marrow of five patients who had a bone marrow aspiration due to neutropenia (median neutrophil counts 2.3 × 10^9^/L), but in whom no hematological disease was established. Each healthy subject was examined to ensure that there was no evidence of bone disease (i.e., osteoporosis, which was excluded using dual-emission X-ray absorptiometry scans of the lumbar spine and the femoral necks or osteoarthritis which was excluded by plain radiography) and no medication that could alter the normal bone turnover during the last 6 months.

#### Data recording and quality assurance

Data were collected from the medical files of the patients. Clinical study monitor performed source data verifications and ensured the accuracy of these data. Among other data we recorded treatment data, treatment outcome according to the International Myeloma Working Group criteria, and patients’ OS.

#### Measurement of Sema4D and Plexin-B1 and of bone markers

For NDMM, serum and bone marrow plasma was collected and stored at the time of diagnosis, before the administration of any kind of therapy. After vein-puncture serum was separated within 4 h and stored at −80 °C until the day of measurement. Similarly, bone marrow plasma was separated within 4 h after bone marrow aspiration and stored at −80 °C until the day of measurement.

Sema4D and Plexin-B1 levels were measured using ELISA methodology (USCN Life Science Inc., Wuhan, China). The minimum detectable dose for Sema4D was less than 0.68 ng/ml; the method has an intra-assay coefficient of variation (CV) of <10% and an inter-assay CV of <12%. The minimum detectable dose for Plexin-B1 was less than 0.59 ng/ml; the method has an intra-assay CV of <10% also and an inter-assay CV of <12%.

In all NDMM patients and controls, we also measured serum CTX (chemiluminescence immunoassay, CTX-I Crosslaps, ImmunoDiagnostic Systems, Herlev, Denmark; sensitivity 0.033 ng/ml, intra-assay CV 1.7–3.0%, inter-assay CV 2.5–10.9%) and bALP (Ostase BAP, ImmunoDiagnostic Systems, Herlev, Denmark; sensitivity 1.0 μg/L; intra-assay CV 1.5–2.7%; inter-assay CV 3.0–6.5%).

#### Evaluation of myeloma-related bone disease

Skeletal surveys using conventional radiography were performed at the time of diagnosis for NDMM patients. A grading of bone morbidity into three stages according to this radiographic evaluation was made: group A included patients with no lytic lesions; group B included patients with 1–3 osteolytic lesions, and group C included patients with more than three osteolytic lesions and/or a pathological fracture due to MM. We have used the <3/>3 as cut-off for bone lesions, as advanced bone disease includes more than three lytic lesions in the Durie–Salmon staging system^20^.

#### Evaluation of MRI pattern of marrow infiltration

T1-weighted (TR/TE: 641/10, turbo factor 4), STIR (TR/TE/TI:2000/70/150), and contrast-enhanced T1-weighted MR images (TR/TE) were obtained in NDMM patients at diagnosis in the sagittal plane for the thoracic spine and for the lumbar spine and in the axial plane for the pelvis with a 1.5 T unit (Phillips Medical Systems, Eindhoven, The Netherlands). MR images were analyzed for pattern of myelomatous involvement. The pattern of marrow involvement on MR images was characterized as: (1) normal when there was no evidence of abnormal signal intensity; (2) focal, which consisted of localized areas of abnormal marrow; (3) diffuse, in which normal bone marrow signal intensity is completely absent; (4) variegated which consists of innumerable small foci of disease on a background of intact marrow.

#### Statistical analysis

Data for continuous variables are presented as mean ± standard error of the mean. Data for categorical variables are presented as numbers and/or percentages. Kolmogorov–Smirnov test was used to test the normality of distribution of continuous variables. Paired *t*-test or Wilcoxon signed-rank tests were used to test for differences within the levels of continuous variables. Independent samples *t*-test or Mann–Whitney test were used for between group comparisons, in cases of two groups of continuous variables. One-way analysis of variance (ANOVA) or Kruskal–Wallis test were used in cases of more than two groups of continuous variables. In case of statistically significant difference in ANOVA or Kruskal–Wallis test, Tukey’s post hoc adjustment was used for multiple pairwise comparisons. Chi-square or Fischer’s exact test were used for between group differences in categorical variables. Spearman’s (rs) coefficient of correlation was used for bivariate correlations between continuous variables. Partial coefficient (*r*_p_) was used for binary correlations adjusted for co-founders. A two-sided *p*-value of less than 0.05 was considered statistically significant in all the above tests. Statistical analysis was performed by SPSS 21.0 for Macintosh (IBM Corp., Armonk, NY).

## Results

### In vitro study: myeloma and ovarian cancer cell lines and Sema4D production

**Fig. 1:**
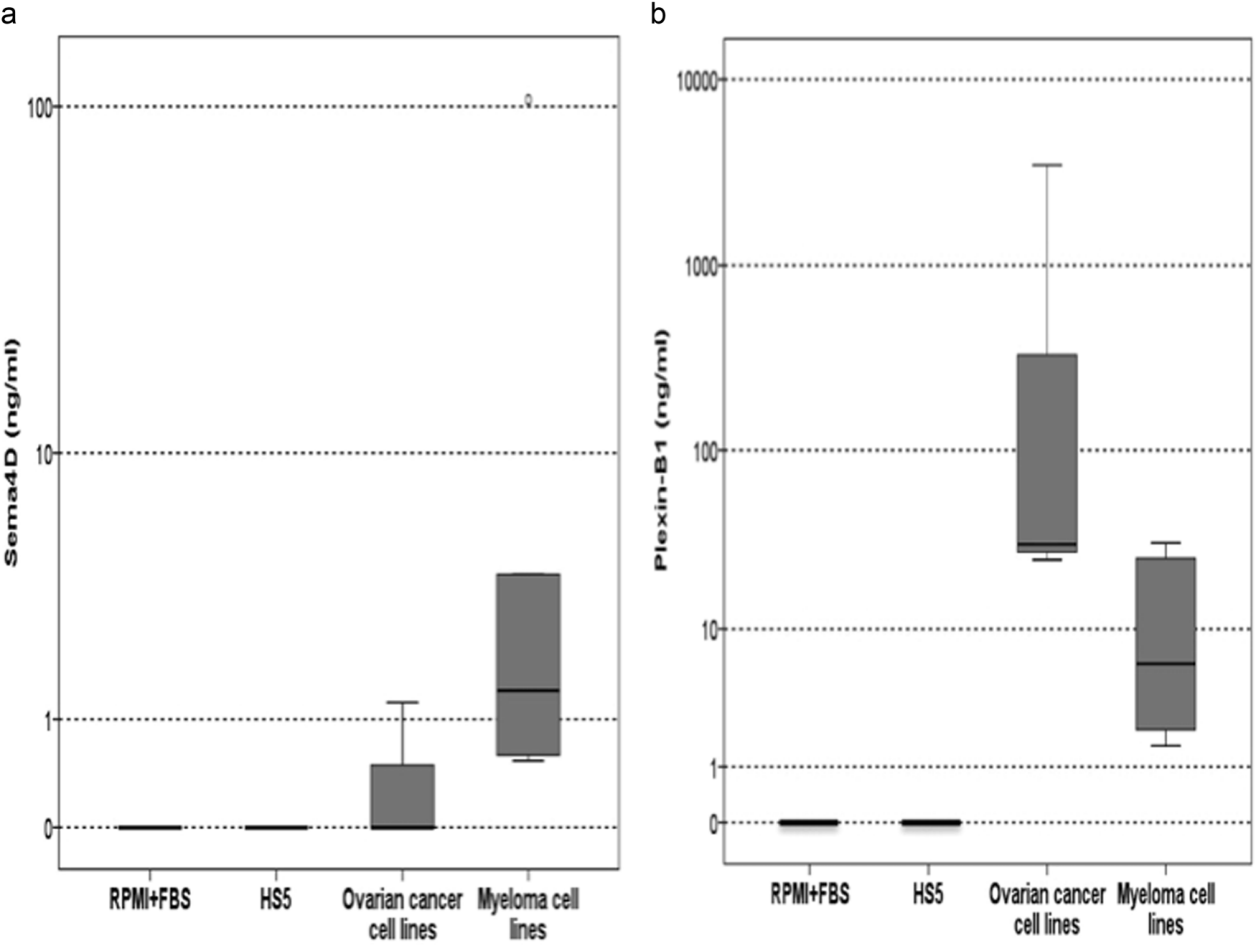
**a** Semaphorin 4D (Sema4D) expression (ng/ml) in RPMI+FBS, HS5 stromal cell line, ovarian cancer, and multiple myeloma cell lines. **b** Plexin-B1 expression (ng/ml) in RPMI + FBS, HS5 stromal cell line, ovarian cancer, and multiple myeloma cell lines (*p* = 0.008)

Only one myeloma cell line produced high Sema4D (MR20: 104.45 ng/ml) compared to all others (mean ± SD: 1.6 ± 1.4 ng/ml), while there were undetectable Sema4D levels in the supernatants of all ovarian cancer cell lines (Fig. 1a). Regarding Plexin-B1, two myeloma cells lines (H929: 25.3 ng/ml and JJN3: 30.8 ng/ml) and two ovarian cancer cell lines (OVCA3: 5125 ng/ml and SKOV3: 3516 ng/ml) produced high levels compared to the other myeloma (4 ± 2.5 ng/ml) and ovarian cancer cell lines (27.6 ± 3.8 ng/ml). The levels of Sema4D and Plexin-B1 in the RPMI + FBS medium were not detectable. The Plexin-B1 levels in the supernatants of the myeloma cell lines (76 ± 140 ng/ml) were decreased compared to the respective levels of the ovarian cancer cell lines (963 ± 1440 ng/ml, *p* = 0.008, Fig. 1b).

**Fig. 2:**
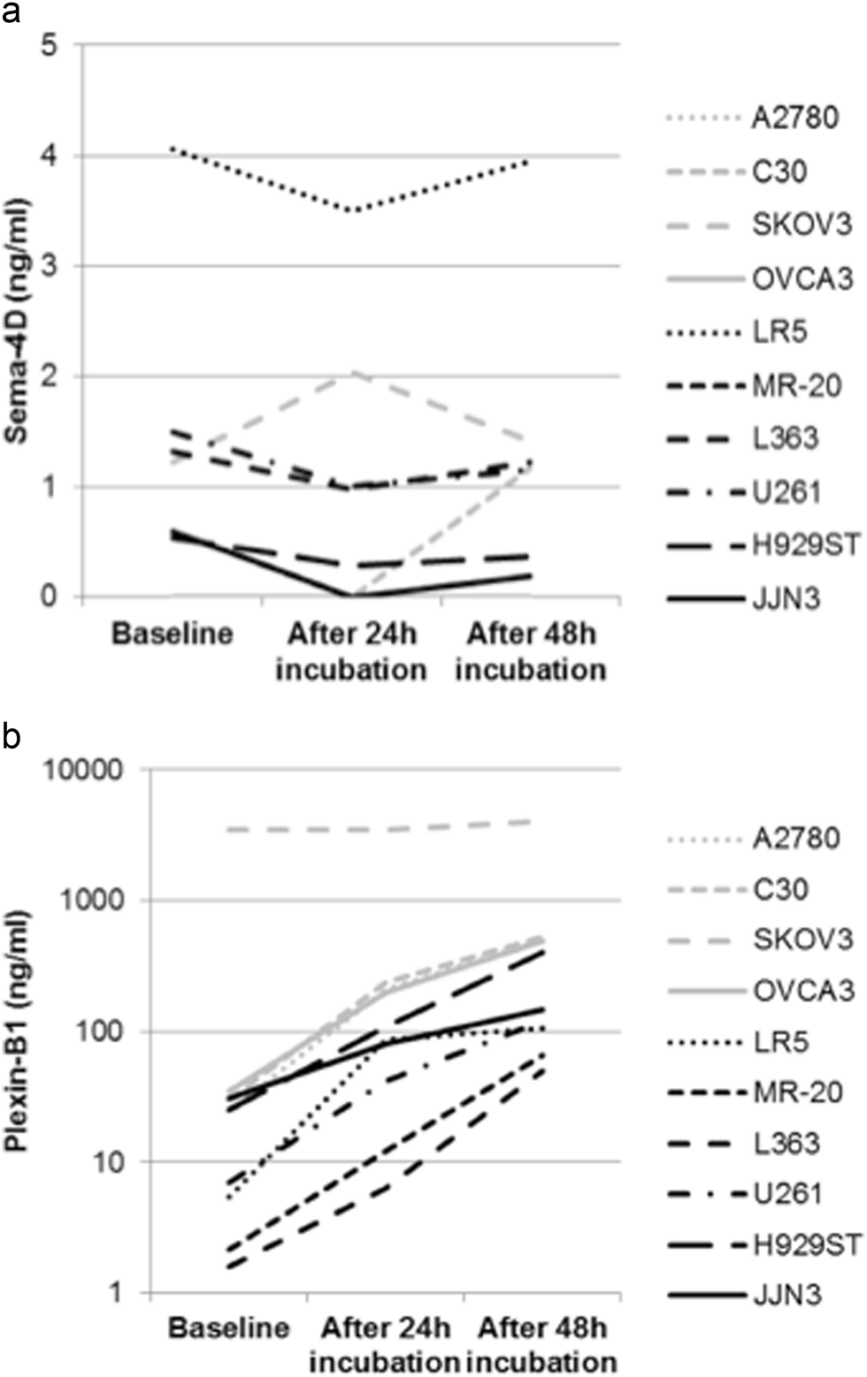
**a** Semaphorin 4D (Sema4D) levels (ng/ml) in the supernatants of ovarian and multiple myeloma cell lines after 24 and 48-h incubation with stromal cell line HS5. **b** Plexin-B1 levels (ng/ml) in the supernatants of ovarian and multiple myeloma cell lines after 24 and 48-h incubation with stromal cell line HS5

After incubation for 24 and 48 h with stromal cell line HS5 (Sema4D and Plexin-B1 levels in the HS5 supernatant were not detectable), there was no alteration in the Sema4D levels of the supernatants of both the myeloma and the ovarian cancer cell lines (Fig. 2a), while there was a 5-fold to 8-fold increase in the Plexin-B1 levels in all studied cell lines except for SKOV3 cell line, where no alteration was noted (Fig. 2b).

### Clinical study

#### Patients

Almost half of the studied patients were male (*n* = 37, 51.4%) with a median age of 70 years. The disease stage per International Staging System was I in 33.3% (*n* = 24), II in 32% (*n* = 23), and III in 34.7% (*n* = 25), respectively. Regarding bone disease staging, 19 patients (26.4%) were classified as stage A, 21 patients (29.2%) as B, and 32 (44.4%) as C, respectively. Elevated serum calcium levels were found in 25 newly diagnosed MM patients (34.7%). MRI pattern of marrow infiltration was focal in 32 (44.4%), diffuse in 25 (34.7%), and normal in 15 (20.8%) patients (Table 1).

**Table 1:** Patient characteristics

Patients (*N*)	72
Gender	
Male	37 (51.4%)
Female	35 (48.6%)
Age	
Median	70
Range	41–88
Ig subtype	
IgG	45 (62.5%)
IgA	17 (23.6%)
BJP	7 (9.7%)
IgD	1 (1.4%)
NA	2 (2.8%)
ISS stage	
I	24 (33.3%)
II	23 (32%)
III	25 (34.7%)
Hb	
<10 gr/dl	30 (41.7%)
Creatinine	
>UNL	20 (27.8%)
≥2 mg/dl	12 (16.7%)
LDH	
>240 U/L	9 (12.5%)
Osteolysis	
Present	53 (73.6%)
Bone disease	
Stage A	19 (26.4%)
Stage B	21 (29.2%)
Stage C	32 (44.4%)
MRI marrow infiltration pattern	
Normal	15 (20.8%)
Focal	32 (44.4%)
Diffuse	25 (34.7%)
Calcium (albumin adjusted)	
≤10.1 mg/dl	47 (65.3%)
10.2–11.5 mg/dl Grade I	19 (26.4%)
11.5–12.5 mg/dl Grade II	4 (5.5%)
12.5–13.5 mg/dl Grade III	2 (2.8%)
>13.5 mg/dl Grade IV	0

*BJP* Bence Jones Proteinuria, *NA* not available, *UNL* upper normal limit, *ISS* Multiple Myeloma International Staging System, *Hb* hemoglobin, *LDH* lactate dehydrogenase, *MRI* magnetic resonance imaging

#### Sema4D and Plexin-B1 in MM patients

**Fig. 3:**
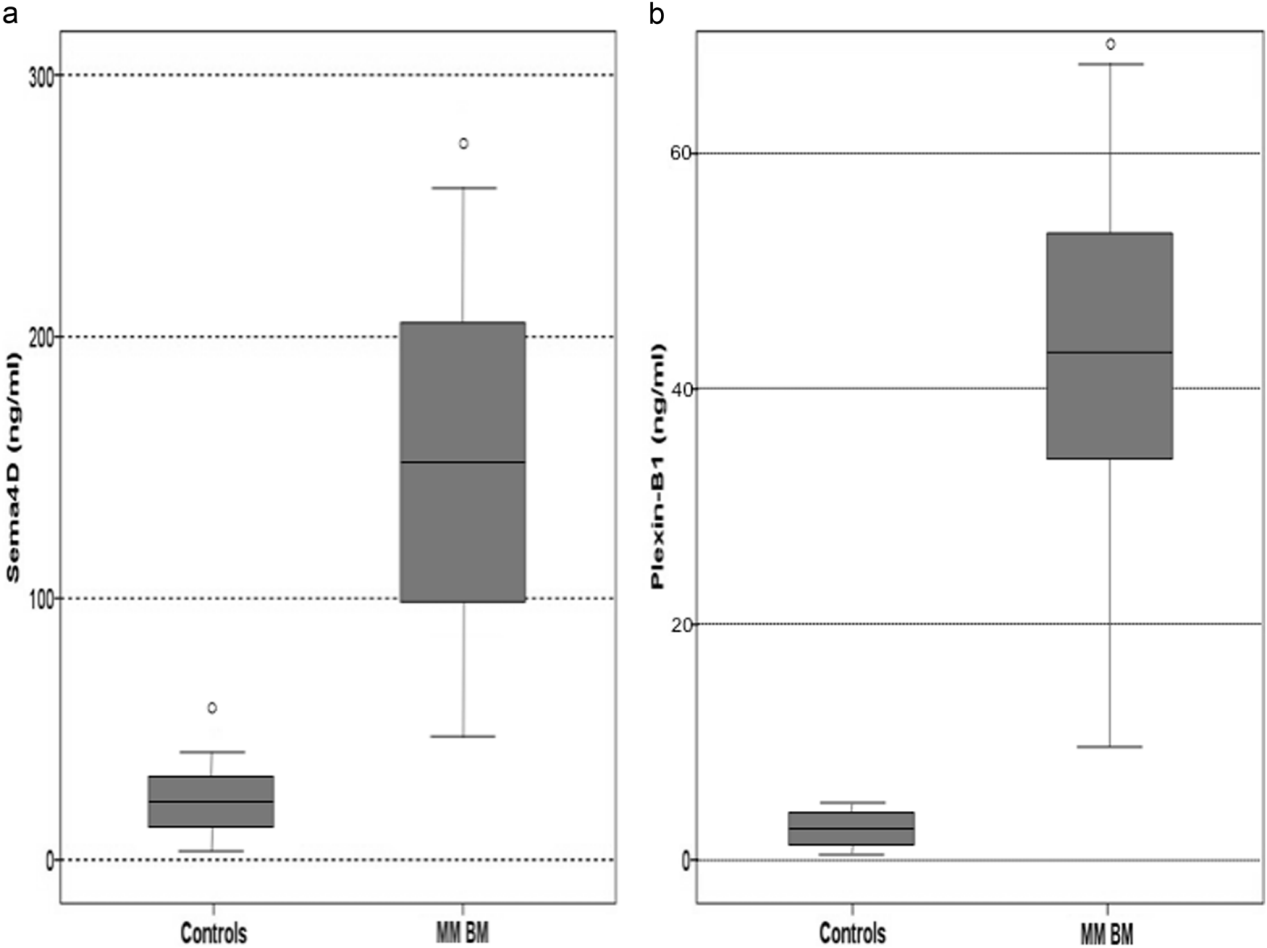
**a** Bone marrow Semaphorin 4D (Sema4D) levels of multiple myeloma patients compared to controls (*p* < 0.01). **b** Bone marrow Plexin-B1 levels of multiple myeloma patients compared to controls (*p* < 0.01)

**Fig. 4:**
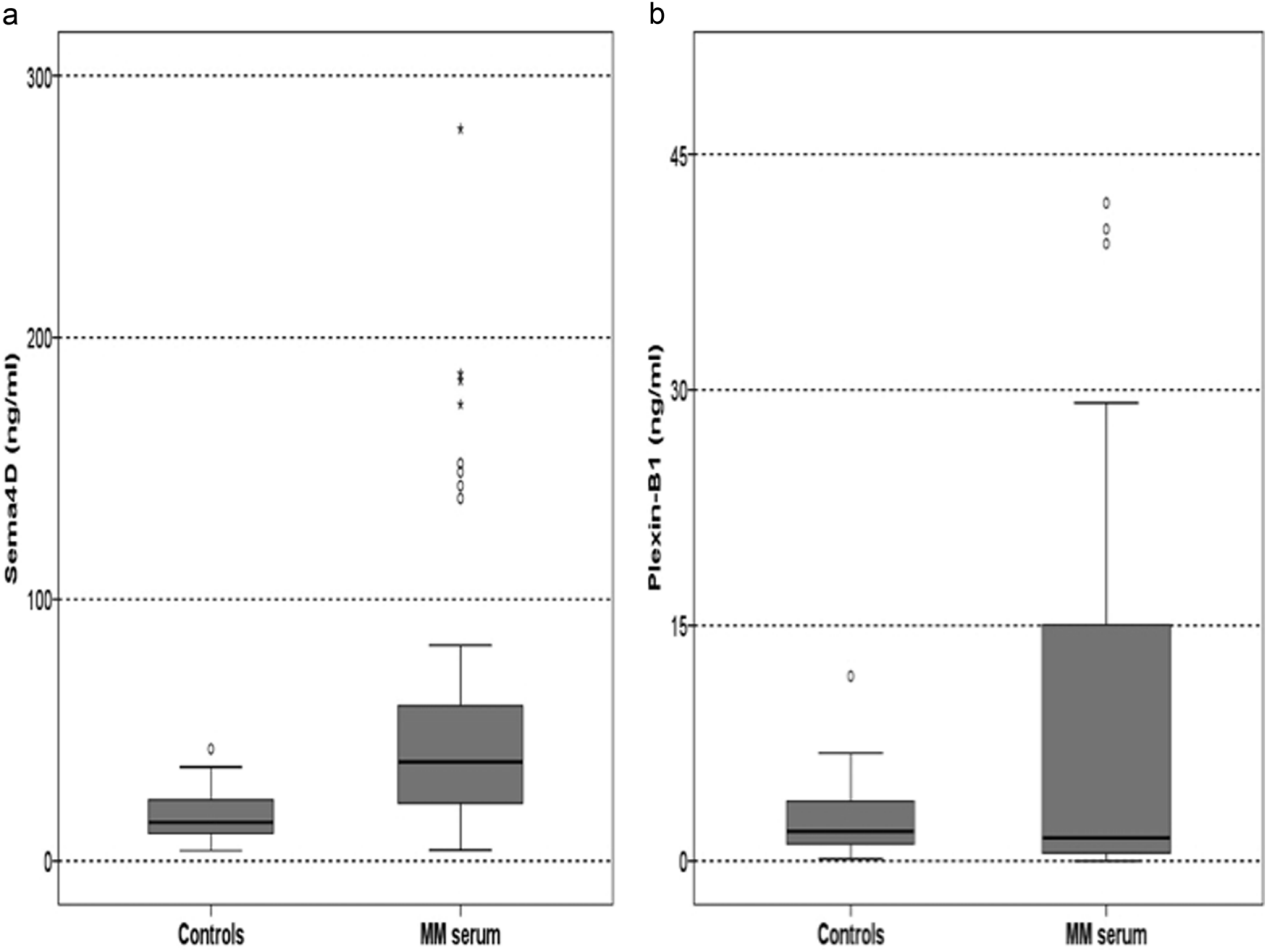
**a** Serum Semaphorin 4D (Sema4D) levels of multiple myeloma patients compared to controls (*p* < 0.001). **b** Serum Plexin-B1 levels of multiple myeloma patients compared to controls (*p* = 0.01)

**Fig. 5:**
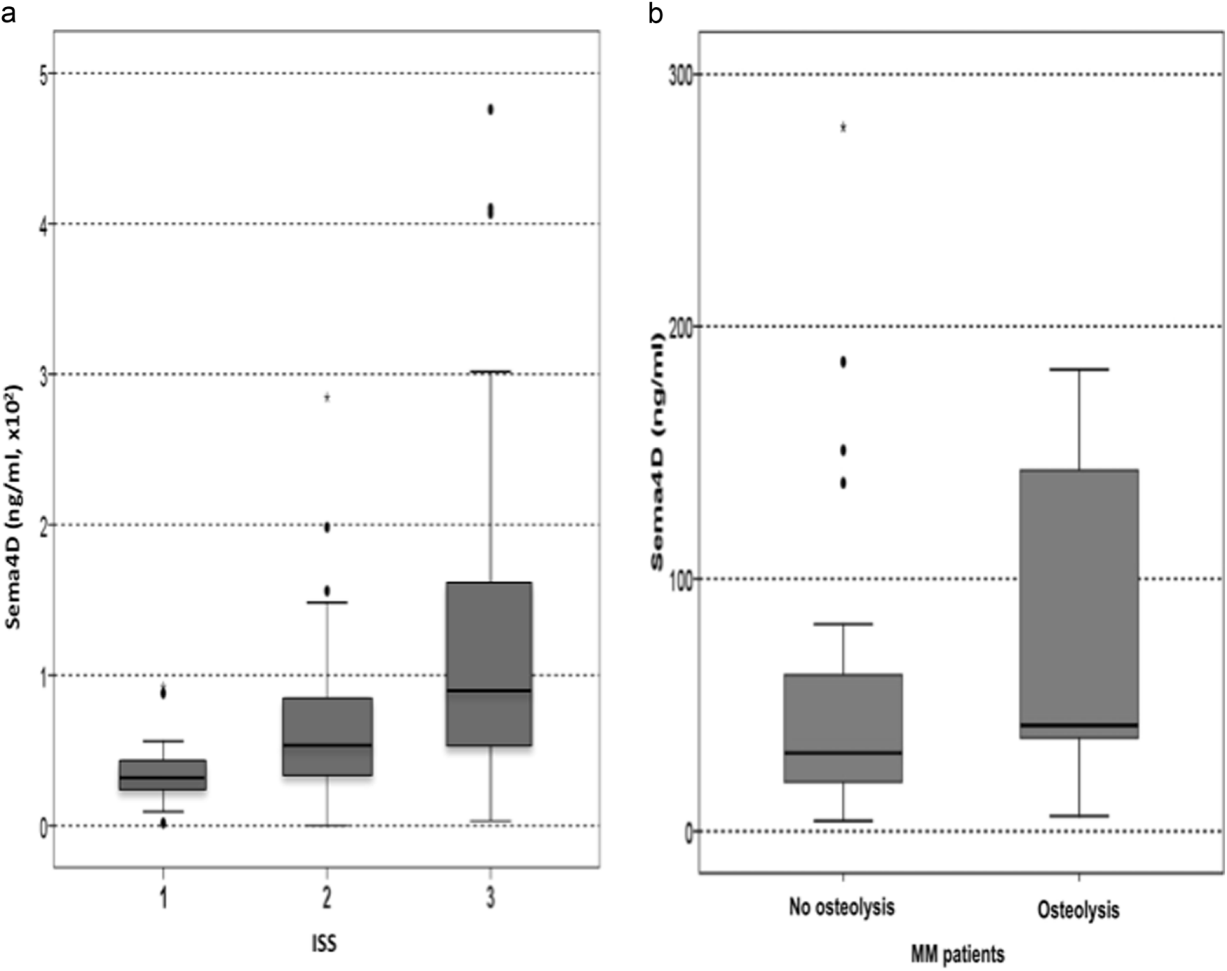
**a** Increased serum Semaphorin 4D (Sema4D) levels correlated with higher ISS stage in multiple myeloma patients (*p* < 0.001). **b** Serum Semaphorin 4D (Sema4D) levels in patients with osteolyses vs. those without bone disease in plain radiographs (*p* = 0.07)

**Fig. 6:**
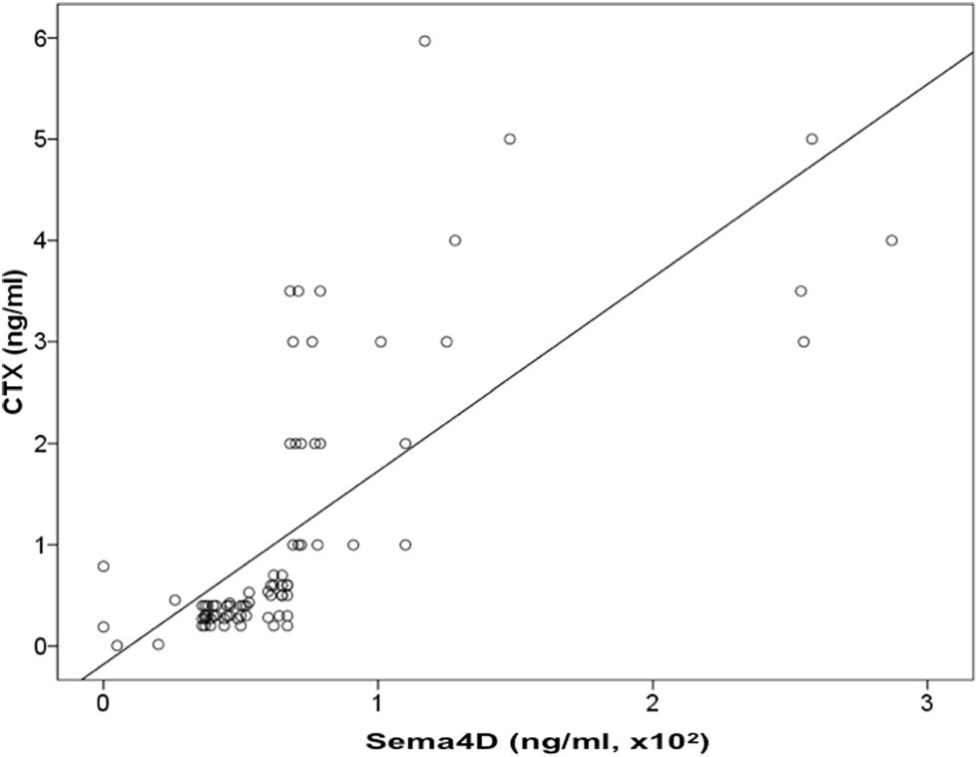
Serum Semaphorin 4D (Sema4D) levels correlated with CTX serum levels (*r* = 0.524, *p* < 0.01) in multiple myeloma patients

The mean Sema4D levels of the bone marrow plasma of the MM patients were dramatically elevated compared to controls (149 ng/ml ± 112 vs. 23 ± 12 ng/ml, *p* < 0.01, Fig. 3a). Similarly, the circulating Sema4D was increased in MM patients compared to controls (71 ± 110 vs. 18 ± 10.9 ng/ml; *p* < 0.001, Fig. 4a). A strong correlation between Sema4D serum levels and bone marrow plasma levels was demonstrated (*r* = 0.628, *p* < 0.001). Sema4D levels in MM patients correlated with serum calcium (*r* = 0.628, *p* < 0.001), ISS stage (ANOVA, *p* < 0.001, Fig. 5a), and CTX serum levels (*r* = 0.524, *p* < 0.01, Fig. 6). Sema4D showed no significant correlation with bALP (*r* = −0.112).

Furthermore, there was a trend for higher Sema4D bone marrow plasma levels in patients with osteolysis in plain radiographs compared to patients without detectable bone disease (*p* = 0.07, Fig. 5b). Patients with diffuse MRI pattern of marrow infiltration had higher levels of bone marrow plasma Sema4D compared to all other patients (161 ± 98 vs. 93 ± 72 ng/ml, *p* = 0.02).

Regarding Plexin-B1, bone marrow plasma and serum levels were increased in myeloma patients compared to controls (44 ± 29 ng/ml vs. 3.4 ± 0.8 ng/ml, *p* < 0.01 and 11 ± 20 ng/ml vs. 2.6 ± 2.7 ng/ml, *p* = 0.01, for bone marrow plasma, Fig. 3b, and serum, Fig. 4b, respectively). There were no strong correlations between Plexin-B1 and other studied parameters.

The median follow-up of the patients was 61 months and the median OS was 46 months. Serum or plasma levels of Sema4D or Plexin-B1 had no impact on patients’ survival. In multivariate analysis, only ISS stage was predictive for survival (HR 3.58, *p* < 0.01).

## Discussion

Bone disease is a hallmark of MM and often results in bone complications. Current therapeutic options for bone complications include bisphosphonates, and more recently denosumab, that has shown promising results in a phase III clinical trial^21,22^. However, bisphosphonates are correlated with jaw osteonecrosis and renal impairment, while they have no effect on osteoblast function. Therefore, the investigation of novel targeted drugs to enhance osteoblast function and improve the management of myeloma-related bone disease is of great interest^23^. Semaphorins are signaling molecules involved in the cell–cell communication between osteoclasts and osteoblasts^6,7^ and especially Sema4D has a dominant effect by suppressing osteoblast differentiation and modulating osteoblast motility^13,14^. Herein, we show for the first time that Sema4D and its receptor, Plexin-B1, are elevated both in the serum and bone marrow plasma of patients with active MM. Our findings are in accordance with the deregulation of bone metabolism in MM characterized by decreased osteoblast activity^1^. In a recent study, evaluating gene expression in skeletal precursor cells after 24 h co-culture with the MM cell line INA-6-altered transcriptome profiles of genes associated with bone metabolism, including Sema4D, were detected^24^. Indeed, MM cells seem to prevent osteoblast differentiation through a Sema4D-mediated mechanism^17^. Moreover, it has been suggested that increased Sema4D levels in the MM–bone niche also attributes to osteocyte-derived Sema4D, apart from osteoclastic production^16^. With regard to myeloma bone disease, we found a trend for higher Semaphorin 4D levels in patients with osteolysis, compared to patients with no evidence of bone disease in plain radiography. This not statistically significant finding is probably due to the number of patients enrolled or due to the role of Sema4D, which takes place within the microenvironment and the circulating forms (both in the plasma and in the serum) possibly do not depict the alterations in the microenvironment. Furthermore, it should be noted that plain radiography is no more the actual standard for assessing myeloma-related osteolytic bone disease. Whole-body low-dose computed tomography might have revealed previously undetected lesions resulting in a different classification of patients regarding the extend of bone disease. This could possibly reveal a more significant correlation with Sema4D levels. On the other hand, the strong correlation of Sema4D with bone resorption marker CTX suggests that Sema4D contributes to bone destruction in myeloma. This finding is further supported by the marked correlation of sema4D with hypercalcemia, which is a result of enhanced bone resorption in MM^25^ and predicts for poor survival in MM patients^26^. Furthermore, the strong association between Sema4D and ISS staging and diffuse MRI pattern of marrow infiltration, which were observed in this study, suggests a correlation of Sema4D with myeloma burden.

Interestingly, we also found a significant correlation between serum and bone marrow plasma Sema4D levels (*r* = 0.628, *p* < 0.001). Sema4D seems not to be produced directly by the myeloma cells, as only one myeloma cell line produced high levels of Sema4D. However, it should be noted that further preclinical research by using myeloma cells lines that may reflect in vivo biology more accurately is deemed necessary in order to reach firm conclusions. Furthermore, its correlation with ISS and diffuse MRI pattern strongly suggest that high Sema4D is produced when myeloma load is also high.

The above results support that Sema4D inhibition might be a promising approach in MM, since preclinical data in other settings have shown positive results. Incorporating a siRNA interference molecule for Sema4D targeting the osteoclasts by a site-specific bone-targeting drug-delivery system in an osteoporotic animal model, induced by ovariectomy, prevented the suppression of osteoblast activity resulting in higher bone volume^27^. Silencing Sema4D expression via RNA interference decreased the number of osteolytic metastases in a breast cancer murine model^28^. Furthermore, antibody-mediated Sema4D blockade showed a promising immunomodulatory effect in murine colorectal and breast cancer models^29^. Targeting Sema4D constitutes a potential novel approach, taking into consideration the limited impact of Sema4D inhibition on bone remodeling, since both osteoclasts and osteoblasts remain intact. Thus, a more favorable toxicity profile may be anticipated^28^.

In conclusion, we demonstrated that the levels of Semaphorin 4D and its receptor Plexin-B1 in both bone marrow plasma and serum are elevated in patients with symptomatic, newly diagnosed MM. These high Semaphorin 4D levels correlate with increased bone resorption, hypercalcemia, and higher ISS stage, and seem to contribute to myeloma-induced bone disease. Further studies will reveal the exact role of Semaphorin 4D/Plexin-B1 signaling pathway in MM pathogenesis, its prognostic significance, and possible future therapeutic implications.
